# Difficulties of Anatomy in America

**Published:** 1830-10-01

**Authors:** 


### XXIX.

### DIFFICULTIES OF ANATOMY IN AMERICA.

WE see by the American journals that our brethren on the other side of the Atlantic labour under the same embarrassment as we do, in regard to dissection. The Massachusetts Medical Society has issued an address to the community, on the necessity of legalizing the study of anatomy, in which they appeal to the feelings—and indeed to the self-interest of their non-professional countrymen, by relating numerous facts illustrating the dire effects of a defective knowledge of anatomy. We hope, and indeed believe, that these illustrations are somewhat too highly coloured—and one of them we grieve to see not only inserted, but, in some degree warranted by our esteemed contemporary, the Journal of Medical Science for May last. We shall quote this melancholy case, and we trust that every thinking reader will agree with us that it is a misrepresentation—an almost palpable impossibility.

"The Address, after some general observations on the indispensable nature of anatomy to the accomplished physician, which are sufficiently familiar to every medical man, goes on to quote several striking instances of the loss of life from patients falling into incompetent hands. An aged practitioner reports more than a hundred persons, under his own observation, dying from strangulated hernia, and the question is very naturally asked, 'how great must have been the number in the whole of New England, who perished miserably from the same cause?' There are also several interesting cases given, somewhat at large, of death from the accidental wounding of large arteries by the ordinary implements of husbandry, and other instruments. There is much good sense in making these statements, because positive instances of evil, are always more readily comprehended than mere abstract argument, and where a question of human misery is concerned, our sympathies are inevitably excited. It has been our misfortune to witness several of those horrors in the practice of surgery, arising from an ignorance of anatomy on the part of operators. If there were no other object in view than to stigmatise an individual, charity would induce us to suppress the narrative, but as an important argument is in question, it is proper to adduce it.

During the brilliant campaign of our army, in 1814, on the Niagara frontier, many cases of severe wounds required surgical operations. A surgeon occupying a distinguished station through his commission, but certainly not through any professional qualification, was a chief operator. We saw this person, in an amputation of the thigh, *fail to cut through the great sciatic nerve; after the bone was sawed through, the limb still hung on by this nerve; ignorant of its nature, he made a plunge at it with his saw, the screams of the poor soldier attested the concentrated agony of a thousand operations, until the operator was implored by an assistant to desist and to use a knife.* A captive officer of the enemy was wounded in the fore-arm, by a musket ball, and from the division of an artery, the bleeding was profuse; several days were spent in attempting to arrest it with a tourniquet. The pressure of the latter at length caused great tumefaction of the limb, and threatened mortification. The same operator instead of taking up the main artery above the wound, amputated the limb, and the operation being performed while it was in a state of inflammation, the pain was immeasurably augmented, and the poor fellow finally fell a victim to the want of scientific skill."

We have put a passage in italics, and we conscientiously believe than an error has crept into the recital. How could the integuments of the thigh be retracted so as to leave the bone bare for the saw, with the great sciatic nerve, and many soft parts between it and the bone undivided? We think the thing impossible! And as to the attempt to *saw* through the *nerve*—it is so absurd, that the most ignorant carpenter would not have dreamt of such a procedure. It is also a great inconsistency to suppose that any man who was a "chief operator," and consequently who must have been able to tie arteries and amputate limbs, should have been entirely ignorant of what a large nerve was when laid bare in an operation. We consider ourselves as DENIZENS of America —and for the honour of that land which has adopted us, we take the liberty of remonstrating with our transatlantic contemporary, and request him to reflect on a passage which casts a deep reflexion on his professional brethren.

